# Supplementary material for: Molecular structure retrieval directly from laboratory-frame photoelectron spectra in laser-induced electron diffraction
Source: Nat Commun. 2021 Mar 9;12:1520. doi: 10.1038/s41467-021-21855-4 (PMC7943781; doi:10.1038/s41467-021-21855-4)
Supplement: Supplementary file 1 — Supplementary Information [file 41467_2021_21855_MOESM1_ESM.pdf]

Supplementary information - Molecular structure retrieval directly from laboratory-frame photoelectron spectra in laser-induced electron diffraction

A. Sanchez[1,*], K. Amini[1,*], S.-J. Wang[2], T. Steinle[1], B. Belsa[1], J. Danek[2], A.T. Le[2,3], X. Liu[1], R. Moshammer[4], T. Pfeifer[4], M. Richter[5], J. Ullrich[4,5], S. Gräfe[6], C.D. Lin[2], J. Biegert[1,7,†]

[1]*ICFO - Institut de Ciencies Fotoniques, The Barcelona Institute of Science and Technology, 08860 Castelldefels (Barcelona), Spain.*
[2]*Department of Physics, J. R. Macdonald Laboratory, Kansas State University, 66506-2604 Manhattan, KS, USA.*
[3]*Department of Physics, Missouri University of Science and Technology, Rolla, MO 65409.*
[4]*Max-Planck-Institut für Kernphysik, Saupfercheckweg 1, 69117, Heidelberg, Germany.*
[5]*Physikalisch-Technische Bundesanstalt, Bundesallee 100, 38116 Braunschweig, Germany.*
[6]*Institute of Physical Chemistry and Abbe Center of Photonics, Friedrich-Schiller-Universität Jena, Helmholtzweg 4, 07743 Jena, Germany.*
[7]*ICREA, Pg. Lluís Companys 23, 08010 Barcelona, Spain.*

[†]*To whom correspondence should be addressed to. Email: jens.biegert@icfo.eu*
**Authors contributed equally.*

Supplementary Note 1: Extraction of field-free scattering information from field-dressed LIED measurements

Supplementary figure 1 illustrates the process of electron rescattering in LIED. Shown is the laser electric (LIED) field (red) and its associated vector potential (yellow, dashed). Due to the higher order of magnitude higher ionization probability for long versus short quantum trajectories, only two long trajectories are shown (blue and turquoise). In MIR-LIED, an electron is tunnel-ionized close to the peak of the electric field. Quantum mechanically, a range of quantum trajectories re-encounter the molecule where the electron scatters, thus acquiring a momentum shift p, which embeds structural information. An analysis of the quantum trajectories[1,2,3] yields that scattering trajectories lead to the highest final (measured) electron kinetic energies, thus allowing to distinguish them from non-scattering trajectories at low kinetic energies. This process repeats every half cycle during the laser field for the range of cycles where ionization occurs. Thus, by tuning the intensity of the laser, the process can be controlled to occur only once. Note that even without such control, the varying (Gaussian) intensity profile leads to a significant modulation of the acquired final kinetic energies, thus permitting to distinguish scattering trajectories even for multi-cycle laser pulses. Another important point is the fact that electron momenta are measured in the laboratory frame. But the described dynamics is governed by the laser field, i.e., the dynamics occurs in the laser polarization frame. A closer look at the dynamics shows that the scattered electron (blue) re-encounters the molecule (distance r=0) at time $t_{r,1}$ in the presence of the laser field, thus acquiring an additional momentum $p(t_{r,1}) = A(t_{r,1})$. The measured momentum consists of two contributions, the momentum which is imparted due to scattering of the molecule k_1 and the momentum imparted by the laser $p(t_{r,1})$. Knowledge about the vector

potential $A(t_{r,1})$ is thus needed to extract k_1 from measuring p_1. This is illustrated by the inset and described in Refs. [4,5].

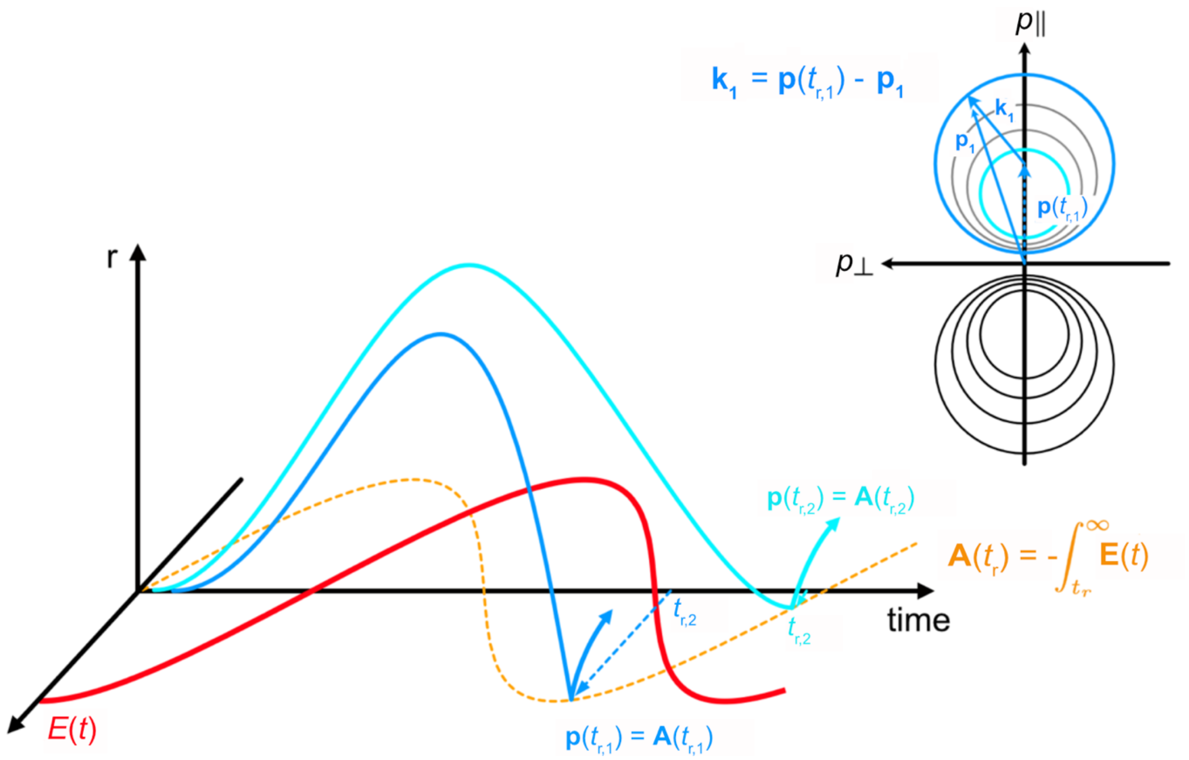

Supplementary Fig. 1 Extraction of field-free scattering signal in the presence of a strong laser field from laboratory-frame photoelectron momentum. Shown is the electric field (red) together with its associated vector potential (yellow dashed) of the LIED laser pulse. An electron tunnel ionizes in the presence of a strong laser field and two possible "long" quantum trajectories are shown in blue and turquoise. The emitted electron is first driven in the direction given by the scalar product $\mathbf{q} \cdot \mathbf{E}$ before returning and scattering against the target at some time later t_r where it acquires momentum transfer k from the target and momentum $p(t_r) = A(t_r)$ from the vector potential. The electron is detected with momentum p.

Supplementary Note 2: Extraction of OCS bond angle and corresponding uncertainty

The OCS bond angle, Φ_{OCS}, was determined by simple geometrical consideration using

$$\Phi_{\mathrm{OCS}} = cos^{-1}\left(\frac{R_{CO}{}^2 + R_{CS}{}^2 - R_{OS}{}^2}{2\, R_{CO} R_{CS}}\right), \tag{1}$$

where the uncertainty in Φ_{OCS} is given by error propagation using a first order Taylor expansion,

$$\delta\Phi_{\mathrm{OCS}} = \sqrt{(\frac{\delta\phi}{\delta R_{OS}}\delta R_{OS})^2 + (\frac{\delta\phi}{\delta R_{CS}}\delta R_{CS})^2 + (\frac{\delta\phi}{\delta R_{CO}}\delta R_{CO})^2}. \tag{2}$$

The area of uncertainty shown in Fig. 4c (of the main text) is extracted by Fourier-transforming the two extrema of both the shaded blue Poissonian error distribution by ΔMCF = $\sqrt{N}/\sigma_{atom}$ and the shaded red detected longitudinal momentum error distribution given by $\Delta q = 2\Delta p_r$.

References

1. Zuo, T., Bandrauk, A. D. & Corkum, P. B. Laser-induced electron diffraction: a new tool for probing ultrafast molecular dynamics. *Chem. Phys. Lett.* **259**, 313–320 (1996).
2. Varró, S. & Ehlotzky, F. A new integral equation for treating high-intensity multiphoton processes. *Il Nuovo Cimento D* **15**, 1371–1396 (1993).
3. Lewenstein, M., Balcou, Ph., Ivanov, M. Yu., L'Huillier, A. & Corkum, P. B. Theory of high-harmonic generation by low-frequency laser fields. *Phys. Rev. A* **49**, 2117–2132 (1994).
4. Blaga, C. I. *et al.* Imaging ultrafast molecular dynamics with laser-induced electron diffraction. *Nature* **483**, 194–197 (2012).
5. Wolter, B. *et al.* Strong-field physics with mid-IR fields. *Phys. Rev. X* **5**, 021034 (2015).
